# An optimized method for extraction and purification of inorganic phosphate from plant material for oxygen isotope ratio analysis

**DOI:** 10.1016/j.mex.2023.102541

**Published:** 2024-01-04

**Authors:** Maria Monrad Rieckmann, Ruth Elaine Blake, Sae Jung Chang, Kristian Holst Laursen

**Affiliations:** a Department of Plant and Environmental Sciences (PLEN), Faculty of Science, Plant and Soil Science Section, Plant Nutrients and Food Quality Research Group, University of Copenhagen, Thorvaldsensvej 40, 1871 Frederiksberg C, Copenhagen, Denmark; b Yale University, New Haven, CT, USA; c Korea Basic Science Institute, Seoul Center, Seoul, South Korea

**Keywords:** Compound-specific, Oxygen, Phosphate, Phosphorus, Plants, Stable isotopes, Phosphate extraction from plants for oxygen isotope ratio analysis

## Abstract

Compound-specific stable isotope ratio analysis of oxygen isotopes in inorganic phosphate can be used to study biological phosphorus cycling and the transformation processes controlling the fate of phosphorus. However, methods for extraction of inorganic phosphate from plant tissue for oxygen isotope ratio analysis are not consistent. Further, the purification into solid silver phosphate can be challenging and laborious. In this work, a detailed and optimized method to provide a more consistent, easily implementable and reproducible extraction using trichloroacetic acid and subsequent purification of inorganic phosphate from plant material for oxygen isotope ratio analysis is presented. Key focus points were: uniform extraction of inorganic phosphate from barley leaves, removal of dissolved organic material, flexibility in regards to the amount of inorganic phosphate extracted for the purification into silver phosphate, reduced use of chemicals and, removal of co-precipitated oxygen-bearing compounds before analysis. Most notable optimizations to the method and associated effects were:

• Drying of plant material before inorganic phosphate extraction increases the method applicability to a broader range of plant sample types.
• Removal of dissolved organic matter improves inorganic phosphate purification.
• Sample volume adjustment according to inorganic phosphate content is vital for effective and quantitative precipitations.

## Graphical abstract

**Figure:**
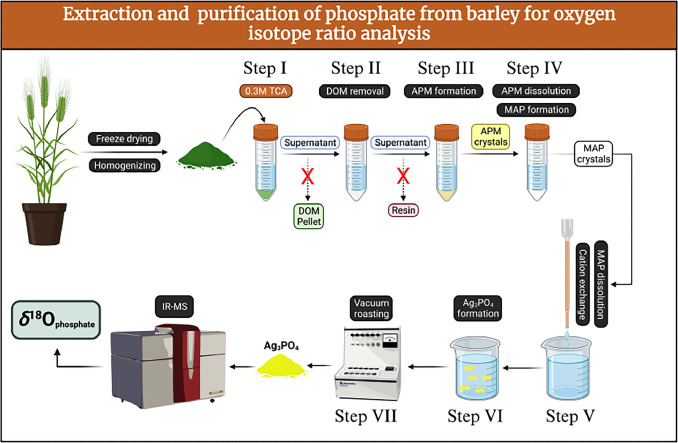

Specifications table

**Table:** 

Subject area:	Environmental Science
More specific subject area:	Phosphate tracing in soil-plant systems
Name of your method:	Phosphate extraction from plants for oxygen isotope ratio analysis
Name and reference of original method:	Pfahler, V., T. Durr-Auster, F. Tamburini, S. Bernasconi, and E. Frossard, 18O enrichment in phosphorus pools extracted from soybean leaves, New Phytologist. 197 (2013) 186-193. https://doi.org/10.1111/j.1469-8137.2012.04379.x
Resource availability:	N/A

## Method details

The optimized phosphate purification method for plant material is adapted and modified from [1], [2], [3] and consists of seven steps (Table 1):

- I: Inorganic phosphate extraction using 0.3 M trichloroacetic acid (TCA)
- II: Resin treatment to remove organic compounds
- III: Precipitation of inorganic phosphate as ammonium phosphomolybdate (APM)
- IV: Precipitation of inorganic phosphate as magnesium ammonium phosphate (MAP)
- V: Cation resin exchange
- IV: Precipitation of inorganic phosphate as silver phosphate (Ag_3_PO_4_)
- VII: Vacuum roasting to remove intracrystalline water and co-precipitated O-bearing compounds

**Table 1:** An overview of the method for extraction and purification of phosphate from barley leaves for oxygen isotope ratio analysis. A short outline of the optimization for each individual step is provided together with the validation method. Phosphate concentrations were measured by ion chromatography (IC).

**Step no.**	**Purpose of step**	**Optimization**	**Validation method**
I	Sample preparation and phosphate extraction.	Extraction was conducted on dry plant material instead of fresh. Extraction was found to be more reproducible for barley leaves and it eliminates the need for cleaning a macerator between samples. Dry plant material can also be stored for longer periods at room temperature.	Comparison of phosphate content measured by IC analysis in 0.3 M trichloroacetic acid (TCA) extracts from fresh versus dry plant material.
II	Removal of dissolved organic matter.	Samples were shaken with DAX-8 resin on an end-over-end shaker to remove dissolved organic compounds as proposed by [3].	The concentration of phosphate was measured by IC analysis before and after resin treatment to assess potential loss of phosphate. Validation was performed on plant material extracted with TCA and on KH_2_PO_4_ dissolved in TCA.
III	Phosphate precipitation as ammonium phosphomolybdate (APM) and dissolution before next step.	The starting volume of the sample was adjusted to the phosphate content per sample as proposed by [1].	After filtration of APM crystals, the filtrate was analyzed by IC for residual phosphate to assess quantitative precipitation.
IV	Phosphate precipitation as magnesium ammonium phosphate (MAP) and dissolution before next step.	Nitrate based magnesia reagent was used to avoid residual chloride in the sample as proposed by [1] The starting volume of the sample was adjusted to the phosphate content per sample as proposed by [1].	After filtration of MAP crystals, the filtrate was analyzed by IC for residual phosphate to assess quantitative precipitation.
V	Removal of interfering cations.	No optimization [4].	The eluent was analyzed by IC for residual phosphate to assess quantitative elution.
VI	Phosphate precipitation as silver phosphate (Ag_3_PO_4_).	The starting volume of the sample was adjusted to the phosphate content per sample, ethanol was used to rinse the beaker to ensure quantitative collection of Ag_3_PO_4_ crystals as proposed by [4].	After filtration of Ag_3_PO_4_ crystals the filtrate was analyzed by IC for residual phosphate to assess quantitative precipitation.
VII	Removal of co-precipitated O-bearing species.	Samples were vacuum roasted to remove intracrystalline water and co-precipitated O-bearing species as proposed by [[5], [6]].	The *δ*^18^O value of certified reference material (Ag_3_PO_4,_ B2207, Elemental Microanalytics, UK) and Ag_3_PO_4_ purified from KH_2_PO_4_ was analyzed before and after vacuum roasting.

Plant material used for development of the optimized method is presented followed by a detailed description of individual steps, including specific optimizations, notes, and observations. This method is solely intended to study oxygen isotopes in TCA-extractable phosphate. In plant studies, the complementary *δ*^18^O value of oxygen isotopes of organic phosphate is highly relevant. For extraction and hydrolysis of organic P the reader is referred to Tamburini et al. [7], where after the present method can be followed from step II.

The solution used to extract inorganic phosphate from plant material was TCA and is found to mainly extract inorganic *ortho*phosphate (PO_4_^3−^) and some sugar phosphates [2,8,9]. Henceforth, the term *phosphate* in this work refers to TCA-extractable inorganic phosphate. Each step was checked for quantitative phosphate transfer for the plant extracts and the reference solution which consisted of pure potassium dihydrogen phosphate (KH_2_PO_4_) with a known *δ*^18^O value dissolved in 0.3 M TCA. The final method (excluding step I) was validated using the reference solution to check for artifacts due to isotopic fractionation. The complete purification method from phosphate extraction to roasting of Ag_3_PO_4_ has a duration of five to six working days for 20-30 samples, depending on available equipment. In the Supplementary Material, a list of chemicals used and protocols for making selected reagents is given.

## Plant material

Barley (*Hordeum vulgare* L., Irina KWS) was germinated in vermiculite for one week and subsequently grown hydroponically for four weeks in a nutrient solution (Table S1 in Supplementary material). The pH was adjusted to 5.5 – 6.0 twice a week using 1 M HCl or 1 M NaOH, and the nutrient solution was changed once a week. The young and old leaves were collected separately, briefly dipped in milli-Q-water and wiped gently with paper towel. Leaves were then divided into two sub samples, one for dry extraction and one for fresh extraction. Barley leaves for extraction from fresh plant material were placed in a plastic bag and stored overnight in a freezer. Barley leaves for extraction from dry plant material were freeze-dried and homogenized in plastic bottles containing zirconium balls in a Retzch mill shaker.

## Step I: sample preparation and phosphate extraction

Extraction of phosphate from fresh barley leaves followed the protocol described by Pfahler et al. [2]. Leaves were added to a container containing 0.3 M TCA, and macerated for 45 s. The sample was placed on an end-over-end shaker at 4°C for 1 h.

Homogenized dry plant material was added to 50 mL PP centrifuge tubes with 0.3 M TCA in a ratio of 1:20 (g:ml) and shaken for 1 h on an end-over-end shaker in a cold room. Samples (fresh and dry extracted) were then centrifuged at 10.000 g for two minutes and the supernatant was filtered through a 0.7 µm GF/F filter (Whatman International Ltd.) into new 50 mL centrifuge tubes using a reusable bottle-top filter (Fisher Scientific Biotech Line). The pelleted residue was washed three times with 2-3 ml of milli-Q water which was also filtered and added to the sample in a new 50 mL tube.

The amount of phosphate (µmol) in the sample was quantified before proceeding with step II. Quantification was conducted by ion chromatography (Dionex™ ICS-2100, Thermo Scientific, Sunnyvale, USA) using Dionex™ IonPac™ AG11-HC (2 × 250 mm) and AS11-HC (4 × 50 mm) columns heated to 30°C, with calibration carried out using a seven-anion calibration standard (Thermo Scientific) and four samples of pure KH_2_PO_4_ dissolved in 0.3 M TCA as a standard with an analytical error of ± 0.2 µmol/L. The limit of detection was determined based on seven injections of the lowest standard and found to be 0.02 µmol/L for phosphate.

### Notes and observations

Due to the leaf structure, fresh barley leaves were difficult to homogenize by maceration, which indicates that this technique might not be applicable to all leaf types/morphologies. The technique is reported to perform well on more soft leaves like soybean leaves [2]. Bauke et al. [10] reported no difficulties during the maceration when leaves of winter wheat were frozen beforehand. Instead of using fresh plant material, as proposed by Pfahler et al. [2], we found that drying the sample before homogenizing, made the extraction more efficient by eliminating the time needed to clean the pestle or macerator between each fresh plant sample. This improved consistency also allowed higher sample throughput.

Solvents most often used to extract phosphate from plant material for oxygen isotope analysis are water [8,11] or 0.3 M TCA [2,[9], [10],^12^,^13^]. It should be noted that a study conducted on eucalyptus by Hawkins and Polglase [8] found that using water or 0.3 M TCA as an extraction solvent for phosphate was equally efficient. Due to the amount of various cations present in leaf material, drying the leaves can potentially lead to free phosphate being precipitated as sparingly soluble compounds such as Ca- and Fe- phosphates and thereby being unavailable for extraction using water or TCA. According to Pons and Guthrie [9], Ca_3_(PO_4_)_2_ is soluble in TCA, thus, it was tested whether the TCA-extractable amount of phosphate was similar between fresh or dry material. The concentrations obtained from the dry leaves were converted to fresh leaf concentrations by adjusting for the water content. When comparing the phosphate concentration extracted from fresh and from dry plant material, no significant difference was observed with concentrations of 56.4 ± 8.4 (average ± SD, n=4) µmol phosphate/g fresh weight and 57.9 ± 13.2 (average ± SD, *n*=4) µmol phosphate/g fresh weight, respectively. Possible precipitation of phosphate by cations during drying was therefore not considered an issue. It is proposed to use dry plant material for a more uniform and easily implementable method.

It was found through sequential extractions of dried homogenized barley material that a sample to solution volume ratio of 1:20 (g:ml) was optimal and is consistent with other studies [2,10,12]. For ease of applying this purification method, at least 15 µmol phosphate should be extracted from the plant material [4]. The lowest amount of phosphate resulting in successful precipitation of Ag_3_PO_4_ is 5 µmol phosphate in a sample [1], yet the purification of such low amounts of phosphate can be problematic when dealing with a complex solution matrix and without thorough removal of dissolved organic matter (DOM).

## Step II: removal of dissolved organic compounds

To each sample, exercised DAX-8 resin (Superlite™, Sigma-Aaldrich) was added to the sample in a 1:5 ml (resin:sample) ratio and shaken for 3 h on an end-over-end shaker in a cold room at 4°C. Samples were then centrifuged at 10.000 g for two minutes and filtered through 0.7 µm GF/F filters (Whatman International Ltd.) into new centrifuge tubes using a reusable bottle-top filter. The resin was washed several times until the sample volume increased by approximately four times, which was sufficient to collect all phosphate from the resin.

### Notes and observations

**Fig. 1:**
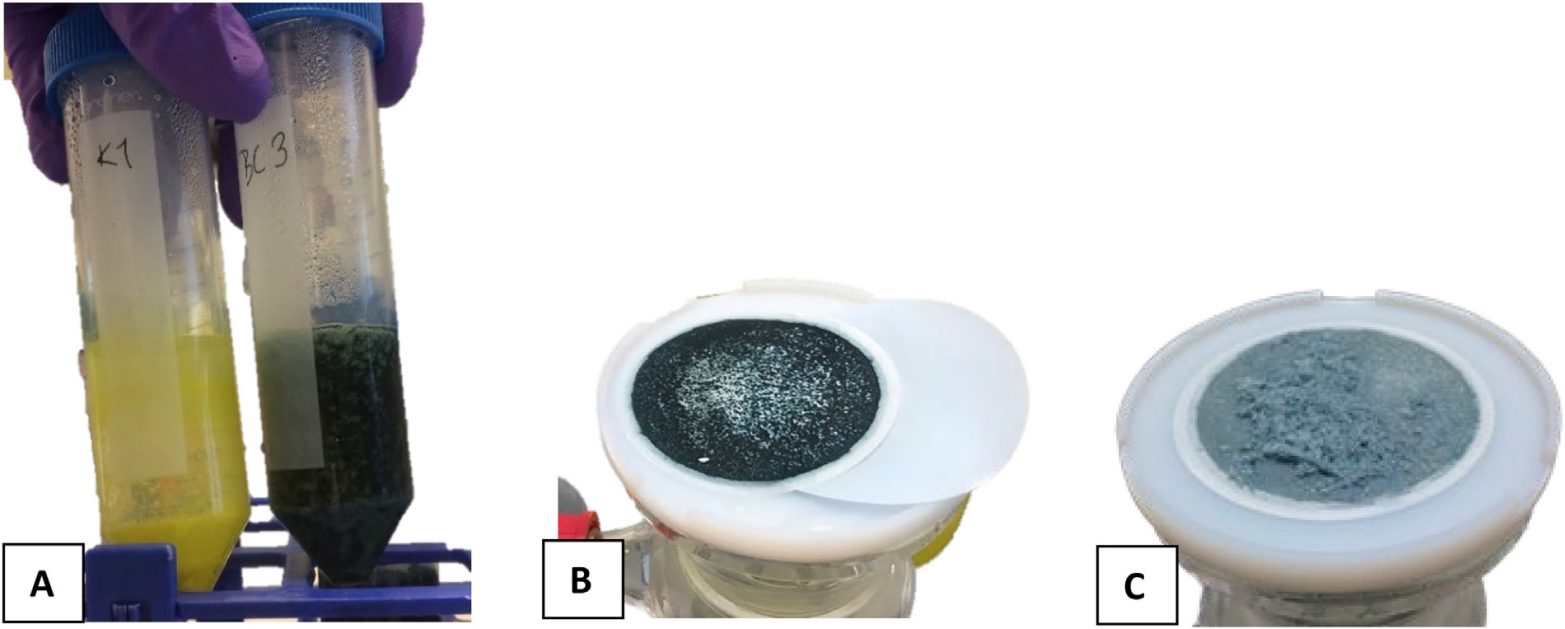
A is an example of the first precipitation step (step III) where phosphate was precipitated as ammonium phosphomolybdate (APM) from pure KH_2_PO_4_ dissolved in 0.3 M TCA (left centrifuge tube) and from a 0.3 M TCA extraction of dried barley leaves without prior DAX-8 resin treatment (right tube). B and C are examples of filtered APM precipitate from a 0.3 M TCA extraction of dried old barley leaves and young leaves, respectively, without a prior treatment with DAX-8 resin (step II).

**Fig. 2:**
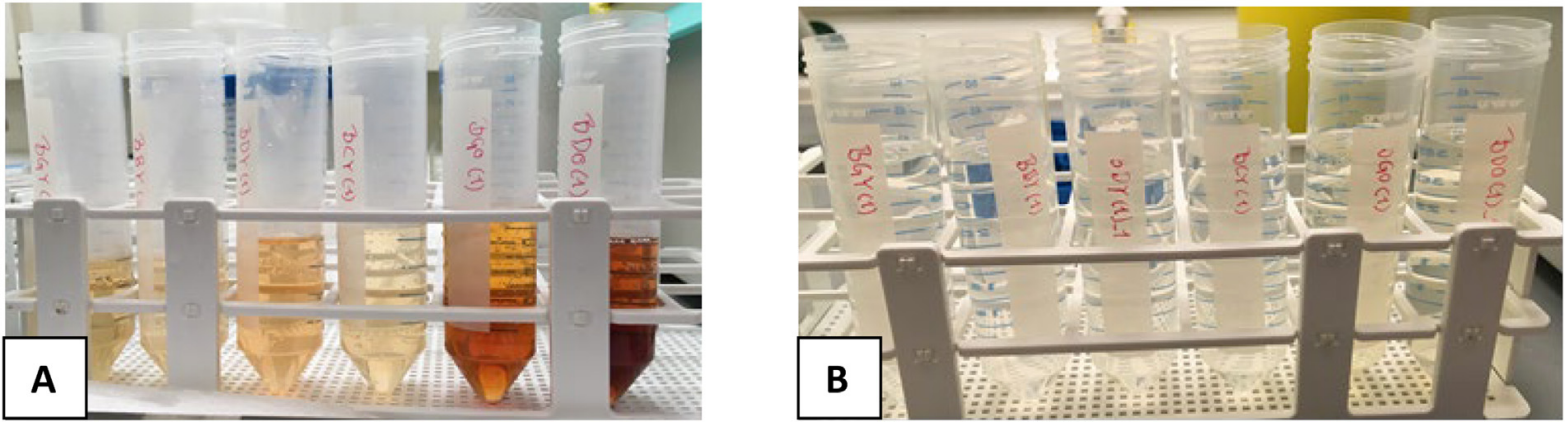
Step II: Tubes containing 0.3 M TCA extracts from young dried barley leaves (first four from the left) or old dried barley leaves (last two) before DAX-8 treatment (A) and after DAX-8 resin treatment (B).

**Fig. 3:**
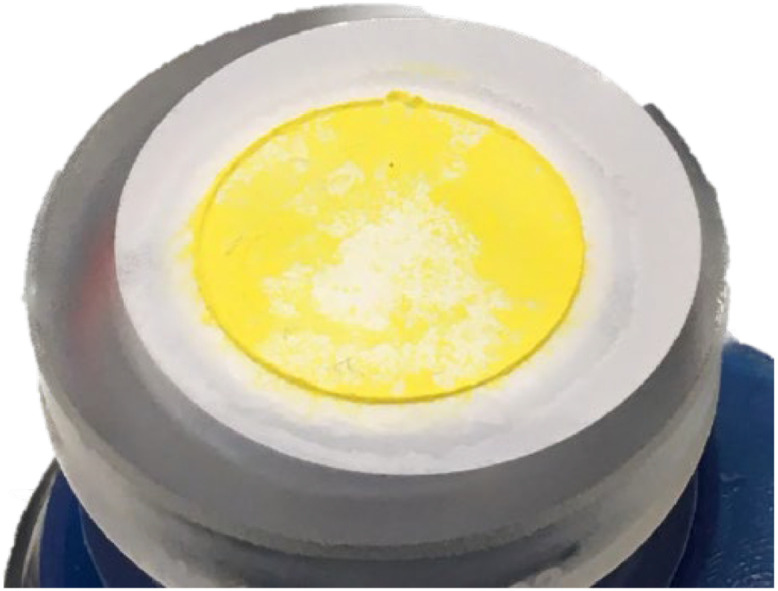
Step III of the purification method showing filtered APM crystals from a 0.3 M TCA extraction on young barley leaves treated with DAX-8 resin before APM precipitation.

It was tested whether the TCA extracted solution should be cleaned for organic co-extractants before APM precipitation. The TCA solution has previously been observed to co-extract some sugar phosphates that may be hydrolyzed by TCA [[2], [9]]. Other non-P containing organic compounds may also be extracted. When phosphate complexes with ammonium molybdate in step III it produces yellow ammonium phosphomolybdate crystals. For samples where co-extracted interfering compounds were not removed, a dark colored sample, usually dark green or blue together with white and dark precipitates, resulted after addition of the ammonium molybdate (AM) reagent to the TCA extract. This indicated complexation and precipitation with ions or compounds other than phosphate (Fig. 1). By including a step that removes organic compounds, in this case the treatment with DAX-8 resin as proposed by [3], the sample became clear and step III proceeded successfully and resulted in yellow APM crystals (Fig. 2, Fig. 3). If the sample continues to be colored after one resin treatment, it is advisable to repeat the resin treatment to remove all interfering compounds.

**Fig. 4:**
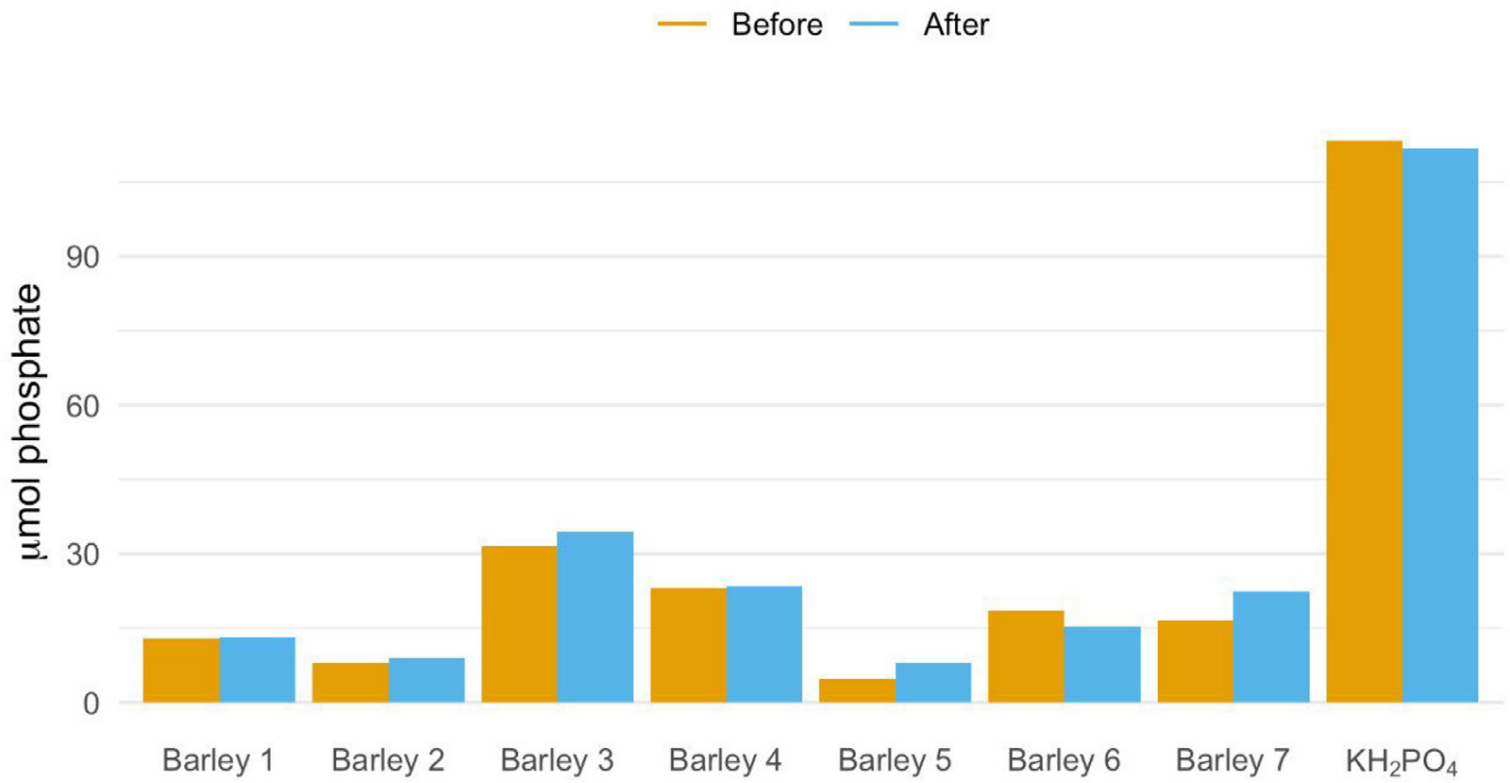
Content of phosphate in 7 individual barley leaf samples extracted with 0.3 M TCA and KH_2_PO_4_ (reference samples) dissolved in 0.3 M TCA. Orange bars show phosphate content before DAX-8 treatment and blue bars are phosphate content after DAX-8 treatment. Phosphate content was measured by ion chromatography.

It was further tested whether phosphate was quantitatively collected from the resin by measuring the phosphate content before and after DAX-8 treatment by ion chromatography. No apparent difference in phosphate content was observed (Fig. 4), which also indicates that no significant amount of dissolved organic P compounds was present and hydrolyzed. It was therefore found that a DAX-8 resin treatment step before starting the first precipitation (step III) was pivotal for a successful outcome.

## Step III: APM precipitation and dissolution

The APM step followed the protocol described by Blake et al. [1] and Colman [14] which were adapted from Tudge [15] and Kolodny et al. [16]. Briefly, to each sample ammonium nitrate (NH_4_NO_3_) in a ratio of 120:1 (NH_4_NO_3_/PO_4_ by weight) was added and samples were swirled until complete dissolution. Subsequently phosphate ions were precipitated as APM crystals by addition of ammonium molybdate (AM) reagent to samples heated to 48°C while mixing. Once APM crystals started to form, samples were left for 10 min and the rest of the AM reagent was added. The total volume of added AM reagent was 7 ml. Samples were left standing overnight to allow for quantitative APM development. Crystals were then recovered using a vacuum filtration system, rinsed with a 5 % NH_4_NO_3_ solution and dissolved in a minimal amount of ammonium citrate reagent into the same centrifuge tube used for the APM precipitation. Samples were centrifuged for 1-2 min to ensure all phosphate containing solution was collected in the bottom of the centrifuge tube for the next precipitation step.

### Notes and observations

The first purification step involves phosphate precipitation from an acidic solution (pH around 1) asAPM using AM reagent. For successful APM precipitation, the volume of the sample should be restricted to below 10 ml for a phosphate sample content of 25-100 µmol, 5-7 ml for a content of 15-24 µmol, and below 5 ml for a content of 5-14 µmol of phosphate [1]. If the sample volume is too large before starting this step, it can be reduced by evaporation at 50-55°C in a fume hood. It is important to take measures to avoid unwanted contaminants in the sample while evaporating. To better stabilize the APM crystals, NH_4_NO_3_ is added to the sample as described by Kolodny et al. [16]. The stabilization makes the precipitation of the crystals faster as more AM reagent was needed before crystallization initiated when no NH_4_NO_3_ was added. Adding NH_4_NO_3_ or not did not affect the δ^18^O value of the final purified Ag_3_PO_4_ as values were *δ*^18^O = 11.1 ± 0.6 ‰ (average ± SD, n=3) and *δ*^18^O = 10.9 ± 0.4 ‰ (average ± SD, n=3) for K_2_HPO_4_ samples dissolved in 0.3 M TCA with and without addition of NH_4_NO_3_, respectively. Also with addition of NH_4_NO_3_, the precipitated crystals were easier to handle as they otherwise had a tendency to act slightly hydrophilic crawling up the sides of the glass funnel. It is essential to collect all crystals as fractionation occurs during precipitation, producing a final isotopically inhomogeneous precipitate with *δ*^18^O values varying over several tenths of a per mil [5]. Other studies have found that if crystal formation does not initiate, it could be due to the pH of the solution or that the conditions of supersaturation were not attained [17]. The TCA solution used for plant material has the optimal pH value of around 1 for APM formation and when restraining the sample volume prior to the precipitation, crystal formation was always successful.

## Step IV: MAP precipitation and dissolution

**Fig. 5:**
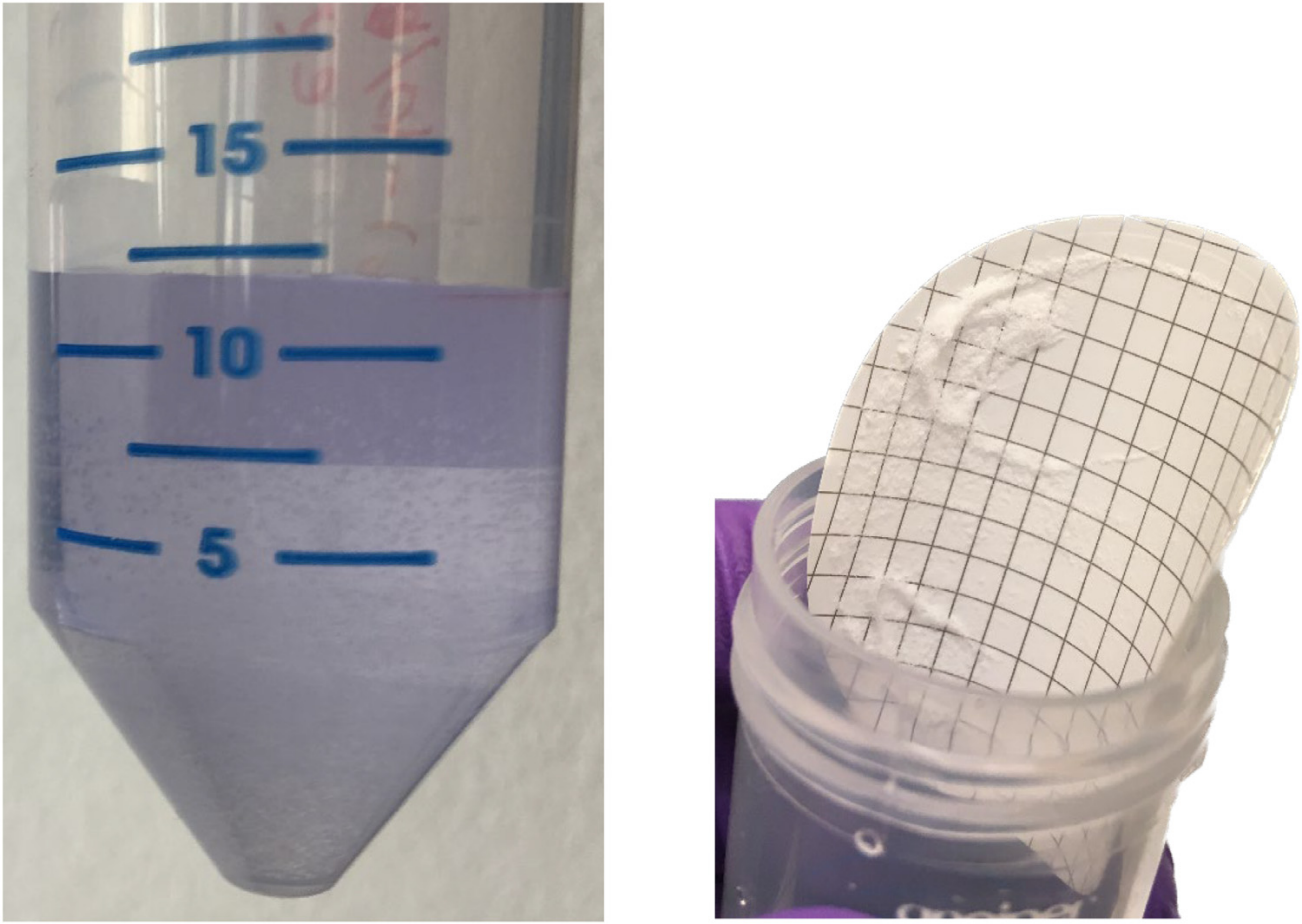
Step IV of the purification method showing formation of MAP crystals (left) from a 0.3 M TCA extraction on young barley leaves and filtered MAP crystals (right).

The MAP step followed the protocol described by Blake et al. [1] and Colman [14] which were adapted from Tudge [15] and Kolodny et al. [16]. Briefly, the sample pH was adjusted to below 7 using nitric acid and MAP reagent was added to give a ratio of 0.25:1 (ml:mg PO_4_). While gently swirling the sample drops of 1:1 (by volume) NH_4_OH were added to increase the pH for precipitation until white MAP crystals were formed (Fig. 5). Usually, crystals were first visible sticking to the side of the centrifuge tube. Then after 20–30 min, an additional 3.5 ml of 1:1 (by volume) NH_4_OH was added to precipitate the remaining MAP. Samples were left at room temperature overnight for quantitative precipitation. Crystals were recovered by vacuum filtration with a glass funnel onto a 0.2 µm nitrate cellulose filter. A 1:20 (by volume) NH_4_OH solution was used to wash the MAP crystals and a minimal amount (preferably below 2mL) of 1 M HNO_3_ was used for the dissolution of the crystals in the same centrifuge tube as used for MAP precipitation (Fig. 5).

### Notes and observations

In this step, phosphate is precipitated with a magnesium reagent as white crystals (magnesium ammonium phosphate) and is achieved at alkaline conditions. The most crucial step in the MAP precipitation is the starting volume [1]. The volume of sample before starting MAP precipitation should be adjusted according to phosphate content. At a too high starting volume the precipitation may take days to occur or not even be successful. It is also advantageous to patiently add the drops of MAP reagents as crystals formed more slowly will be larger and the risk of losing very small crystals during subsequent filtration is avoided. As observed by Blake et al. [1] successful precipitation occurred at a starting volume below 1.5 ml for 5-10 µmol, below 2 ml for 11-15 µmol, below 4 ml for 16-25 µmol, below 5 ml for 25-50 µmol and below 7 ml for 50 -100 µmol phosphate of sample before MAP started to precipitate. For samples containing below 10 µmol phosphate only add 1 ml 1:1 NH_4_OH before leaving samples overnight [1].

In some more recent studies [2,3], the magnesium reagent used for the MAP precipitation step was made with chloride-based reagents. However, chloride can cause problems in the final precipitation step, as chloride competes with phosphate for the silver and can form AgCl_2_ precipitates.

## Step V: cation exchange step

**Fig. 6:**
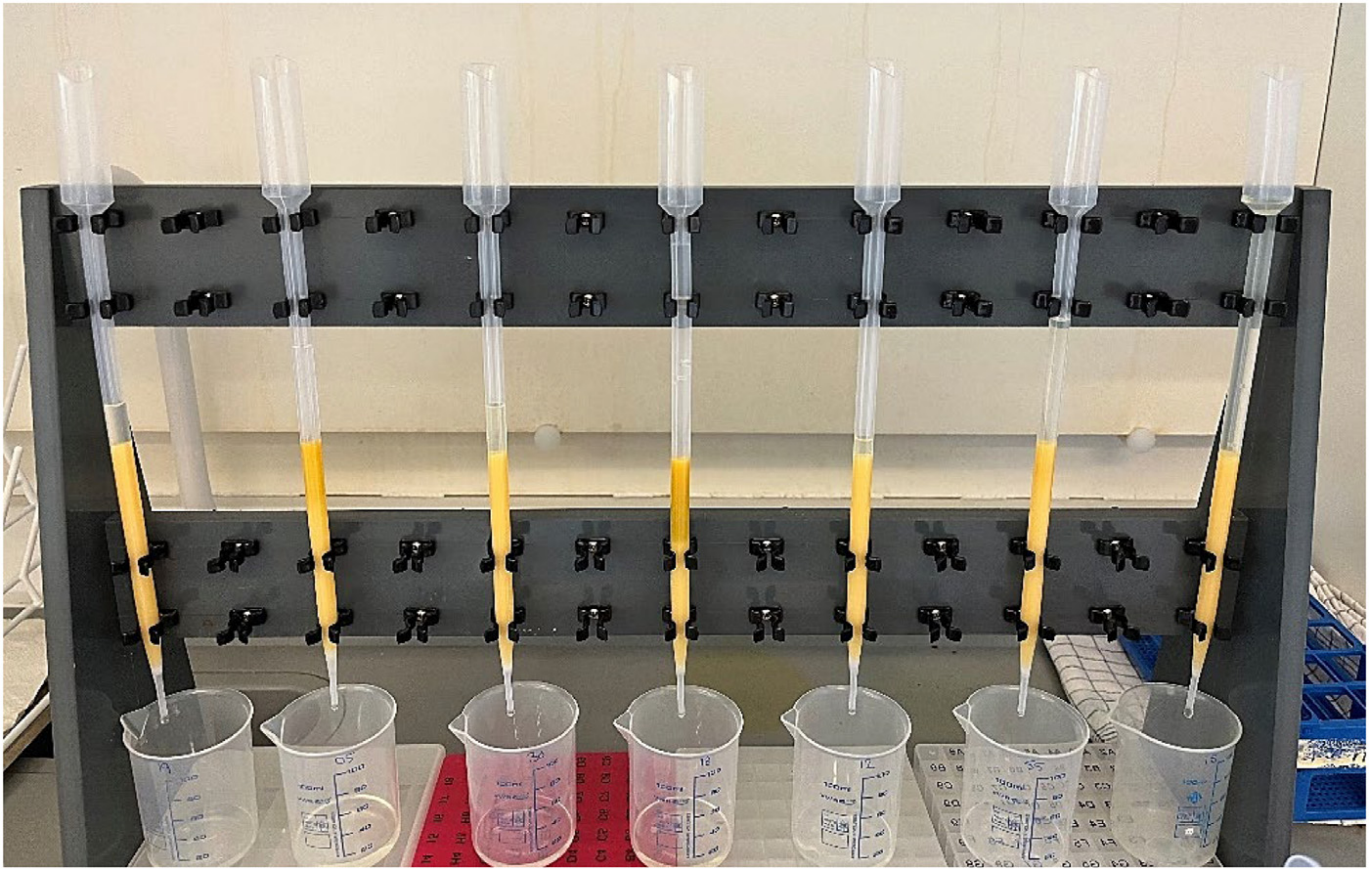
Step V of the purification method showing an example of the cation exchange column setup. Transfer pipettes (15 ml) were plugged with a small amount of glass wool and a large hole cut in the top. Rinse water was collected in small beakers, which were replaced with acid washed plastic beakers once the sample had been added to the resin column.

The cation exchange step followed the protocol described by Liang [4] and Blake et al. [1]. From the MAP dissolution step the sample was strongly acidic due to the HNO_3_, so the pH was adjusted to 5 by 1M NaOH before starting the cation exchange step. Transfer pipettes with a capacity of 15 ml were cut on the top and the tip plugged with quartz wool. Transfer pipettes were filled with 4 ml exercised resin slurry (Bio-Rad, AG50W-X8, 100-200 mesh, H^+^-form) and placed in a rack (Fig. 6). Following sample elution, the cation resin was rinsed carefully with milli-Q water to collect all dissolved phosphate.

### Notes and observations

It is important to perform a cation exchange step to remove cations that could interfere with the final precipitation of silver phosphate, like residual Mg^2+^ or Na^+^ ions [18]. The cation exchange step can be performed in column or batch mode [3,4,17] as long as all counter ions are removed properly by allowing enough equilibration time with the resin and the phosphate is quantitatively collected. This column setup provided a satisfactory flowrate (ml/min). The step can be conducted during a working day, and the final precipitation step can be started on the same day. To reduce the amount of time used on the column step, or simply if the phosphate content is in the low range, the amount of rinse water can be reduced by monitoring for residual eluting phosphate by the molybdenum blue method [19]. Rinsing of the resin can stop once all the phosphate has eluted.

## Step VI: silver phosphate precipitation

**Fig. 7:**
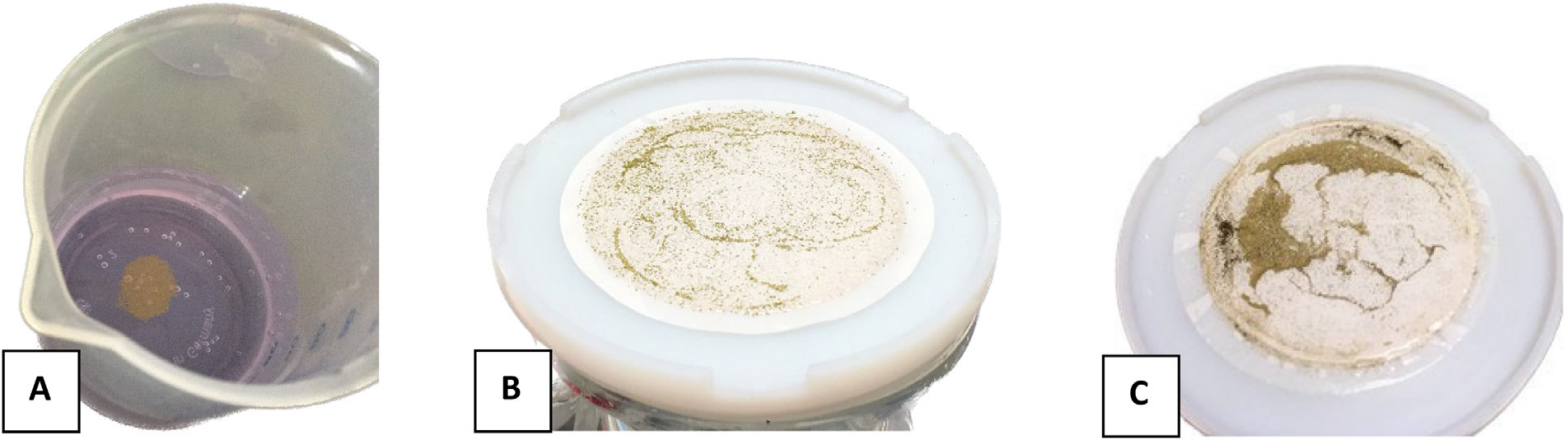
Step VI of the purification method showing silver phosphate crystals formed from phosphate extracted from barley leaves (A). Filtered silver phosphate crystals purified from KH_2_PO_4_ dissolved in 0.3 M TCA (B) and from phosphate extracted from barley leaves (C). Co-precipitated organic material is visible in the silver phosphate purified from barley leaves (dark spots).

Phosphate was finally precipitated as Ag_3_PO_4_, following the protocols described by Colman [14] and Liang [4] which were adapted from Crowson et al. [20] and O'Neil et al. [5]. The pH of the sample was raised to around 10 by dropwise addition of 1:1 (by volume) NH_4_OH and 0.85 ml of silver ammine buffer solution was added per 15 µmol phosphate in the sample. The sample was then placed at 60°C in the dark until yellow Ag_3_PO_4_ crystals were formed (Fig. 7). Once crystal formation was completed, samples was removed from the heat and crystals were filtered onto a 0.2 µm polycarbonate filter using a vacuum filter flask system. The beaker was rinsed with milli-Q water several times to collect as many crystals as possible. If crystals were adhering to the side of the beaker, ethanol was added to the beaker and it was sonicated until all crystals were released (usually 1-2 min). The ethanol and remaining crystals were transferred onto the filter and the beaker was washed thoroughly with milli-Q water. The funnel was released from the support without releasing the vacuum and held above the filter. A transfer pipette was used to carefully wash any crystals adhering to the funnel down onto the filter on the support. The filter containing the crystals was transferred into a glass petri-dish and left at 60°C overnight (or until completely dry). Once the crystals were dry, they were homogenized on weighing paper using a pestle.

### Notes and observations

Silver was added to the sample as a silver ammine buffer solution under alkaline conditions and elevated temperature. Ammonia is evolved from the solution resulting in decreasing pH. Silver starts complexing with phosphate to create yellow crystals at around pH 7 [5,18]. Before precipitation is initiated, the sample volume should be adjusted according to the phosphate content for successful precipitation (20 ml for 15-20 µmol, 30 ml for 21-30 µmol, and 45 ml for higher phosphate concentrations). For concentrations below 10 µmol see the method of micro-precipitation by Blake et al. [1]. It was found preferable to use plastic containers rather than glass for the precipitation of silver phosphate crystals. Crystals precipitated in glass seemed to stick more to the walls, and full quantitative removal was difficult. If the precipitation is conducted overnight, the temperature can be reduced to prevent the sample volume from decreasing to less than a 1/4 of the starting solution as this might initiate precipitation of silver oxide species (Ag_2_O or AgOH). The lower the temperature applied during crystal formation, the larger the crystals will be as the formation is slower, making them easier to handle. When the crystals are dry, they must be completely homogenized before analysis due to potential fractionation upon crystal formation [5].

## Step VII: removal of unwanted co-precipitates

Homogenized crystals were transferred to HPLC glass vial inserts and put into quartz tubes and vacuum roasted at a pressure of 20 mTorr and a temperature of 400°C for 1 h using a Vacprep (Micromeritics).

### Notes and observations

**Fig. 8:**
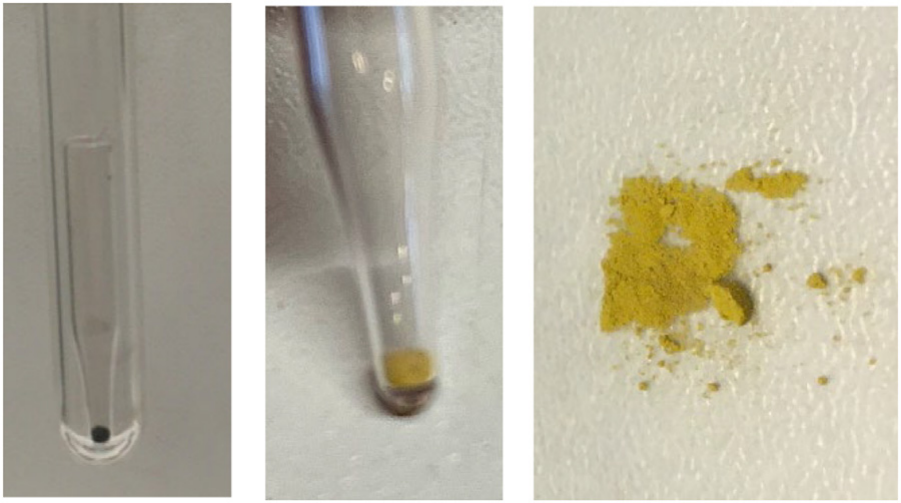
Step VII of the purification method showing purified phosphate as silver phosphate from barley leaves before vacuum roasting (left) and after vacuum roasting at 400°C, 20 mTorr for 1 h (middle and left).

Even though the sample in the beginning of the purification method, after the DAX treatment, seems clean (a clear solution), some dissolved organic compounds may still be present. Co-precipitated organic contaminants were sometimes observed in samples coming from plant material (Fig. 7). These contaminants may be O-bearing, and together with potentially co-precipitated silver oxide species, might bias the true oxygen isotope ratio value of phosphate. Different methods to remove residual organic contaminants have been proposed like reaction with hydrogen peroxide or vacuum roasting [3,21,6]. Applying hydrogen peroxide can take up to several days [3], whereas vacuum roasting the crystals is fast and can be achieved in 3 min per sample at 550°C [22], however, lower temperatures can be used for longer reaction times [6,23]. These conditions are sufficient remove to organic contaminants and intracrystalline water [6,22,24] (Fig. 8). Roasting efficiency and unwanted fractionation during the roasting step was investigated by vacuum roasting pure certified reference material (CRM) of Ag_3_PO_4_ and pure CRM of Ag_3_PO_4_ mixed with an aliquot of dry plant material followed by oxygen isotope ratio analysis. No difference in the measured *δ*^18^O was detected between the two samples (data not shown).

## Oxygen isotope ratio analysis of silver phosphate

Samples of 0.3 to 0.32 milligrams of Ag_3_PO_4_ were weighed and packed into silver capsules and loaded into a PYRO Cube Elemental Analyser (Elementar, Hanau, Germany) in pyrolysis mode. Delta values, *δ*^18^O, were measured by an Isoprime100 isotope ratio mass spectrometer (IRMS) - (Isoprime100, Elementar, Manchester, UK), and reported with respect to Vienna Standard Mean Ocean Water (VSMOW). *δ*^18^O values were calibrated by analysis of certified reference materials of Ag_3_PO_4_ (B2207, Elemental Microanalytics, UK, *δ*^18^O = 21.7) and IAEA-601 (IAEA-International Atomic Energy Agency, Vienna, Austria, *δ*^18^O = 23.3), and drift and linearity was assessed by analysis of an internal standard of Ag_3_PO_4_ (Tribasic, 98 %, Merck). To validate the optimized purification protocol, the reference sample (KH_2_PO_4_) was carried through the purification method several times and the oxygen isotopic signature of the purified Ag_3_PO_4_ was compared to that of KH_2_PO_4_ yielding *δ*^18^O values of 10.9 ± 0.4 ‰ (average ± SD, n=14) and *δ*^18^O =11.3 ± 0.5 ‰ (average ± SD, n=12), respectively. There was no statistically significant difference between the two values (*t*-test (p < 0.05)).

### Notes and observations

To assess analytical quality parameters such as drift and linearity an O-bearing internal standard should be used. Before acquiring the in-house Ag_3_PO_4_ that was used as internal standard in this study, a pure BaSO_4_ was used. However, during analysis, a decline in the *δ*^18^O value of CRM Ag_3_PO_4_ through the analysis sequence was observed when using BaSO_4_. It was suspected that residual barium in the pyrolysis tube did potentially bind to released phosphate during pyrolysis resulting in inadequate release of O because Ba_3_(PO_4_)_2_ dissociates at a much higher temperature of 1850°C [25].

From the isotope ratio analysis, the total content of oxygen per sample can be calculated and can be used to assess the yield of the purification method. However, if co-precipitated species are present during vacuum roasting, there may be residual elements present after roasting (e.g. elemental silver), which may give slightly inaccurate total oxygen yield of the sample. This is mostly important for samples containing very small amounts of phosphate as the ratio of residual elements to phosphate may be high. Yields of phosphate extracted from plant material were between 95 and 99 %.

## Summary of conclusions

In the present study an optimized extraction and purification method to obtain TCA-extractable inorganic phosphate as Ag_3_PO_4_ for oxygen isotope ratio analysis from plant material was presented. Barley leaf material was dried and homogenized before phosphate extraction as it gave a more standardized and reproducible extraction. Thorough removal of organic matter in the sample before the first precipitation (step III) is crucial for the subsequent purification. For a successful outcome in each step, the sample volume needs to be adjusted according to the phosphate content. After the final precipitation of phosphate as Ag_3_PO_4_ a vacuum roasting step is required to remove co-precipitated O-bearing compounds.

## Ethics statements

No ethical considerations were required.

## CRediT authorship contribution statement

**Maria Monrad Rieckmann:** Methodology, Investigation, Validation, Writing – original draft, Visualization. **Ruth Elaine Blake:** Methodology, Supervision, Writing – review & editing. **Sae Jung Chang:** Methodology, Writing – review & editing. **Kristian Holst Laursen:** Supervision, Conceptualization, Writing – review & editing.

## Declaration of competing interest

The authors declare that they have no known competing financial interests or personal relationships that could have appeared to influence the work reported in this paper.

## Data Availability

Data will be made available on request.

## Appendix Supplementary materials

Supplementary material associated with this article can be found in the online version References Blake, R.E., S.J. Chang, and A. Lepland, Phosphate oxygen isotopic evidence for a temperate and biologically active Archaean ocean, Nature. 464 (2010) 1029-U89. https://doi.org/10.1038/nature08952 Pfahler, V., T. Durr-Auster, F. Tamburini, S. Bernasconi, and E. Frossard, 18O enrichment in phosphorus pools extracted from soybean leaves, New Phytologist. 197 (2013) 186-193. https://doi.org/10.1111/j.1469-8137.2012.04379.x Tamburini, F., S.M. Bernasconi, A. Angert, T. Weiner, and E. Frossard, A method for the analysis of the delta O-18 of inorganic phosphate extracted from soils with HCl, European Journal of Soil Science. 61 (2010) 1025-1032. https://doi.org/10.1111/j.1365-2389.2010.01290.x Image, application 1
