# Supplementary material for: An optimized method for extraction and purification of inorganic phosphate from plant material for oxygen isotope ratio analysis
Source: MethodsX. 2024 Jan 4;12:102541. doi: 10.1016/j.mex.2023.102541 (PMC10823127; doi:10.1016/j.mex.2023.102541)
Supplement: Supplementary file 1 — Supplementary material associated with this article can be found in the online version References Blake, R.E., S.J. Chang, and A. Lepland, Phosphate oxygen isotopic evidence for a temperate and biologically active Archaean ocean, Nature. 464 (2010) 1029-U89. https://doi.org/10.1038/nature08952 Pfahler, V., T. Durr-Auster, F. Tamburini, S. Bernasconi, and E. Frossard, 18O enrichment in phosphorus pools extracted from soybean leaves, New Phytologist. 197 (2013) 186-193. https://doi.org/10.1111/j.1469-8137.2012.04379.x Tamburini, F., S.M. Bernasconi, A. Angert, T. Weiner, and E. Frossard, A method for the analysis of the delta O-18 of inorganic phosphate extracted from soils with HCl, European Journal of Soil Science. 61 (2010) 1025-1032. https://doi.org/10.1111/j.1365-2389.2010.01290.x [file mmc1.docx]

SUPPLEMENTARY MATERIAL

**1. Chemicals used in the purification method**

Stock solutions: ammonium nitrate (ACS, Sigma-Aaldrich ≥ 98 %), trichloroacetid acid (EMSURE® ACS, Reag. Ph Eur, Sigma-Aaldrich), molybdenum(VI) trioxide (ACS reagent, ≥ 99%, Sigma-Aaldrich), ammonium hydroxide (ACS reagent, 28.0-30.0 % NH_3_ basis, Sigma-Aaldrich), nitric acid (67-69 %, PlasmaPure, SCP science), citric acid monohydrate (ACS, Sigma-Aaldrich), magnesium nitrate hexahydrate (ACS reagent, 99%, Sigma-Aaldrich), silver nitrate (ACS, Sigma-Aaldrich), sodium hydroxide (Sigma-Aaldrich), methanol (LC-MS grade, Thermo Fisher Scientific).

**2. Protocols for selected solutions used in the purification protocol**

2.1 AM reagent for APM precipitation

Transfer 25 g MoO_3_ to a glass beaker. Add 100 ml Milli-Q water and 20 ml concentrated NH_4_OH. Heat the solution until the MoO_3_ is completely dissolved. It may be necessary to keep this solution hot while performing the next step but minimize evaporation. In a glass bottle containing a magnet add 150 ml milli-Q water and slowly add 100 ml 70 % HNO_3_. Place a glass funnel in the acid solution with the tip of the funnel just beneath the solution. Slowly pour a small aliquot of the AM reagent through the funnel and let the white precipitate dissolve before adding more. Leave the solution at room temperature for 48 hours before use. If precipitate forms, do not use the solution.

*2.2 Ammonium citrate solution*

In a fume hood, dissolve 10 g of citric acid in 300 ml milli-Q water and 140 ml concentrated NH_4_OH (28-30%) in a 500 ml PE plastic bottle.

*2.3 Magnesia reagent for MAP precipitation*

Add approximately 400 ml milli-Q water to a 500 ml HDPE/LDPE bottle and dissolve 31.5 g of MgNO_3_ and 74.8 g NH_4_NO_3_ in the water**.** Add 10 ml NH_4_OH and leave the solution overnight**.** Then acidify the solution using 9 ml HNO_3_. Pour the solution into a 500 ml volumetric flask and fill up with milli-Q-water.

*2.4 Ag-ammine buffer for 15-120 µmoles of phosphate*

Transfer 17.0 g AgNO_3_ to a HDPE amber wide mouth bottle containing 125 ml milli-Q water and add 25.5 ml concentrated NH_4_OH to dissolve the AgNO_3_. Then add 14.0 g NH_4_NO_3_ and additional 334 ml milli-Q water.

Table S1. Stock nutrient solutions. One ml of each stock solution was added per L double deionized water for the hydroponics setup (5 L nutrient solution per bucket with 4 plants in each). The nutrient solution was changed once a week. All stock solutions were was made in milli-Q water.

| \| **Stock** \| **Chemical** \| **Concentration** \| \| --- \| --- \| --- \| \| **solution** \| **composition** \| **(mol/L)** \| \| A \| KH_2_PO_4_ \| 0.2 \| \|  \| K_2_SO_4_ \| 0.2 \| \| B \| MgSO_4_·6H_2_O \| 0.3 \| \|  \| NaCl \| 0.1 \| \| N \| Mg(NO_3_)_2_·6H_2_O \| 0.3 \| \|  \| Ca(NO_3_)_2_·4H_2_O \| 0.9 \| \|  \| KNO_3_ \| 0.6 \| \| Micro \| Fe(III)-EDTA-Na \| 0.05 \| \|  \| MnCl_2_·4H_2_O \| 0.001 \| \|  \| ZnCl_2_ \| 0.0007 \| \|  \| CuSO_4_·5H_2_O \| 0.0008 \| \|  \| H_3_BO_3_ \| 0.0019 \| \|  \| Na_2_MoO_4_·2H_2_O \| 0.0008 \| |
| --- | --- | --- | --- | --- | --- | --- | --- | --- | --- | --- | --- | --- | --- | --- | --- | --- | --- | --- | --- | --- | --- | --- | --- | --- | --- | --- | --- | --- | --- | --- | --- | --- | --- | --- | --- | --- | --- | --- | --- | --- | --- | --- | --- | --- | --- |
